# A Method for Determination of the Transmission Efficiency of a Silica Optical Fiber Cable Using a Solar Power Tower

**DOI:** 10.3390/ma15041511

**Published:** 2022-02-17

**Authors:** Luís Guerra Rosa, Guilherme De Almeida, José Carlos Garcia Pereira, Alejandro Martínez-Hernández, José González-Aguilar

**Affiliations:** 1 IDMEC—Instituto de Engenharia Mecânica, Instituto Superior Técnico, University of Lisboa, Av. Rovisco Pais, 1049-001 Lisboa, Portugal; guilhermedealmeida@clix.pt; 2 Departamento de Engenharia Química, Instituto Superior Técnico, Universidade de Lisboa, Av. Rovisco Pais, 1049-001 Lisboa, Portugal; jose.carlos.pereira@tecnico.ulisboa.pt; 3 High Temperature Processes Unit, IMDEA Energy, Avda. Ramón de la Sagra 3, 28935 Móstoles, Spain; alejandro.martinez@imdea.org (A.M.-H.); jose.gonzalez@imdea.org (J.G.-A.)

**Keywords:** silica optical fibers, concentrated solar energy, radiation transmissivity, acceptance angle, numerical aperture

## Abstract

After being adequately captured and concentrated, solar radiation can be conducted by optical fiber bundles/cables and directly used for illumination (lighting) or heating of confined spaces, or indirectly used by converting it in other forms of energy (e.g., for producing electricity). This article reports preliminary tests conducted on a 7-m-long optical fiber bundle/cable with an effective aperture circular area of 14 mm in diameter, specially designed and manufactured by a leading company to transmit up to 1000 W_th_ of unfiltered concentrated sunlight. The cable was tested in the typical receiver position at the top of a solar concentration central tower. The main purpose was the experimental determination of the transmission efficiency of the cable in function of the incidence angle using selected groups of heliostats belonging to the heliostat field. The testing methodology proved to be capable of evaluating the performance of the cable. The cable withstood the tests without revealing any type of damage. The results obtained showed that the transmissivity of the cable is higher than 50% when the incidence angle of the solar radiation is lower than 14.7°, increasing sharply to circa 95% when the incidence angle is lower than 4.5°.

## 1. Introduction

As important contributors to limit CO_2_ emissions in industrial or domestic activities, solar technologies can be developed for a variety of uses [1,2]. Nowadays, the technologies to harness solar energy are usually categorized into direct solar-to-electricity conversion and solar-to-heat conversion technologies. The first category refers to photovoltaics (PV), and the second includes low-temperature solar thermal (i.e., solar heating and cooling, SHC) and medium and high-temperature solar thermal (i.e., concentrating solar power (CSP)). CSP uses optical systems to concentrate solar light and leads to applications such as electricity generation, solar thermochemistry, and industrial process heat. Considering the classification used for solar heat for industrial processes (SHIP) [3], low temperature SHIP are in the range of 80–150 °C; medium temperature SHIP cover the 150–400 °C range; and high temperature SHIP are considered to cover the 400–1500 °C range.

The rapid development of solar-driven high-temperature technologies for (solid) materials processing at the industrial scale is becoming urgent. Despite the tremendous potentialities of the usage of solar heat for materials processing, one of the main current shortcomings is the attainment of a homogeneous temperature distribution on the processed materials, due mainly to the unidirectionality of the radiation pattern and the non-homogeneous distribution of the highly concentrated radiant flux. Thus, there is a need for innovative systems for capture, concentration, control, and conduction of concentrated solar radiation, and it is foreseen that most of the new systems will take advantage of the capabilities and benefits of the usage of (advanced) optical fibers, allowing the transmission of solar radiation to locations difficult to get to [4,5,6]. If concentrated solar radiation can be conducted by an optical waveguide composed of low-loss optical fibers, then new devices will certainly be developed, and the lack of the temperature homogeneity will be overcome.

Optical fibers could direct the concentrated solar radiation to the place of utilization and illuminate the target from more than one single direction. Recent works on computer light modelling and analysis have shown that theoretically a quasi-homogeneous solar flux distribution can be obtained by means of a double reflection using two paraboloid reflecting surfaces [7,8]. This is relevant also for the case of optical fiber bundles/cables because it is recommended that the energy flux be smoothly distributed over the irradiated area at the entrance of the bundle/cable, i.e., a flux distribution as homogeneous as possible is ideal.

Application of optical fibers to the transmission of solar radiation was reported by Kato and Nakamura as early as 1976 [9]. They studied the theoretical ability of transmission of solar radiation by optical fibers and found that with fused-silica-core optical fibers it is possible to obtain an average attenuation corresponding to approximately 25 dB/km, i.e., about 6 per cent loss after a 10 m long path. In their paper, they mentioned that “possible areas of application of this relatively short transmission length include the connections and integration of components of solar energy utilization systems such as photovoltaic cells, solar thermal power generation systems, and solar heating, cooling and illuminating systems” [8].

Then, in 1981, Cariou et al. [10,11] suggested attaining long range transmission of concentrated solar energy via optical fibers (TCSEvOF), but, owing to the unavailability of high-quality optical fibers and the high cost of their design, their project scope was limited to a theoretical analysis [12,13]. A review on systems for TCSEvOF was presented by Kandilli and Ulgen [12]. Experimental works addressing high fluxes were supported by using parabolic dishes. In 1998, experimental results of flexible fiber optic solar energy transmission of 60 W using a 19-fiber bundle were reported by Liang et al. [14]. In 2002, Feuermann et al. [15] described experimental tests in which fluxes of about 11 kW/m^2^ were transmitted by optical fibers to a remote target. Additionally, the implementation of optical fibers in concentrated tower systems has been proposed by several authors, e.g., [16,17,18].

An innovative optical waveguide solar power system was constructed and firstly presented in 2009 by Nakamura et al. [19,20,21]. It consists of a double-reflection device to obtain a low-angle incident beam, which is essential for the use of optical fiber bundles to conduct concentrated solar radiation, because it is necessary to satisfy the acceptance angle (typically < 14°) of the optical fiber cables [22]. More recent research aims to produce various flux distributions in 2D and 3D space through corresponding optical fiber bundle arrangements [23]. However, contrary to high-temperature applications like those envisaged by Nakamura, most of the current studies deal with illumination and hybridization with PV systems (e.g., [24,25]) or even with photocatalytic wastewater treatment (e.g., [26]) or photocatalytic CO_2_ reduction [27], and, to our knowledge, no experimental demonstration has been carried out yet assuming the bundle of optical fibers in the receiver position on top of a high-concentration central tower (as was envisaged by González et al. [18]).

The present work reports for the first time the efficiency of an optical fiber cable/bundle (specially designed to transmit concentrated sunlight) using a heliostat field. As the incidence angles required for this work are relatively small (ranging from 0° to 30°) only groups of two or three selected heliostats were used for each experiment, resulting in relatively low radiation-flux concentrations (the maximum attained value was circa 145 kW/m^2^).

This manuscript is organized as follows: First we present some theoretical considerations related to the principles of fiber optics (Section 2); then we explain the experimental setup including the tested fiber cable (Section 3). Results are reported and discussed in Section 4, and final conclusions are summarized in Section 5.

## 2. Efficiency of Optical Fibers for Sunlight Transmission

Flexible optical fibers are fabricated by drawing to a small diameter a preform containing a core and a cladding with decreasing refractive indexes of silica (SiO_2_) glass or of a plastic polymer. Plastic fibers are usually made of polymethyl methacrylate (PMMA), polystyrene (PS), or polycarbonate (PC) [28]. Depending on their characteristics, optical fibers can be used for different applications: telecommunications (especially advantageous for long-distance communications), sensors (for example, to measure strain, temperature, pressure), light guides (e.g., endoscopes, illumination), and also for power transmission to convert the light into heat. Silica fibers are more expensive than plastic fibers, but for solar light applications, they show low attenuation coefficients and excellent resistance to high temperatures (glass transition temperature of vitreous silica is considered higher than 1000 °C), and the fibers can be protected by special heat-resistant coating materials (e.g., [29]). Moreover, silica fibers can withstand solar radiation fluxes as high as 28,000 kW/m^2^ [30], corresponding to 28,000 suns (1 sun is defined as 1000 W/m^2^). Recently Roy et al. [26] obtained experimentally the spectrum of the sunlight delivered through a silica optical fiber bundle and concluded that the spectrum shows good agreement with the natural sunlight spectrum.

Table 1 compares some relevant characteristics of silica glass, polymethyl methacrylate (PMMA), polystyrene (PS), and polycarbonate (PC). The maximum service temperature of a given material is usually provided by the manufacturer, and it can be defined as “the highest temperature at which the material can reasonably be used without oxidation, chemical change or excessive deflection or creep becoming a problem” [31]. Factors like degree of purity of the material and the environmental conditions may strongly affect the maximum service temperature.

Data summarized in Table 1 show that silica glass withstands exceptionally high temperatures and presents an extremely low (~0.55 × 10^−6^/°C) coefficient of thermal expansion (CTE). Due to its very low CTE, silica glass is adequately resistant to thermal shock and thermal cycling. Being a glass, vitreous silica softens progressively as it approaches the melting point of cristobalite (1713 °C) [33]. In an oxidizing atmosphere, the maximum recommended working temperature is 1050 °C, though it may be taken to 1350 °C for short periods; above this temperature, surface devitrification to cristobalite occurs [33]. It should be noted that, using heat-resistant hermetic coatings, silica fibers can be further developed to become protected against oxidizing and moisture environments [29,34].

Presently, silica fibers with hermetic carbon layer are commercially available with core diameters in the range between 100 to 600 μm [35]. The lower the diameter of a fiber, the lower is the minimum allowable bending radius because mechanical flexibility of a fiber increases when its diameter decreases. To preserve great flexibility and simultaneously increase the capacity of transmission of solar light, thousands of individual fibers are bundled together, thus forming a fiber cable. To understand the fundamentals of the work herein reported, it is essential to know how light is reflected and transmitted inside an optical fiber, and how these mechanisms depend on the refractive indexes of the optical media involved.

**Figure 1:**
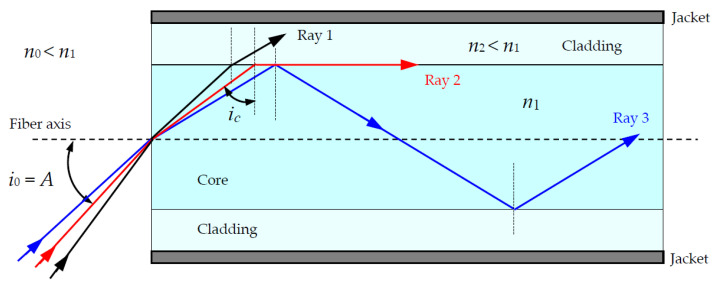
Path of three light rays inside an optical fiber. Colors are only illustrative for schematic purpose.

Each optical fiber in the bundle/cable acts as an independent light waveguide. An individual optical fiber typically includes a core surrounded by a transparent cladding material with a lower index of refraction (see Figure 1). Light is kept in the core by the phenomenon of total internal reflection, which causes the fiber to act as a waveguide. As shown in Figure 1, there are three different possibilities for light rays entering the fiber, represented by Ray 1, Ray 2, and Ray 3. Ray 1 is not transmitted, and it is lost in the cladding. Ray 2 is at the limit of total internal reflection. The symbol *A* represents the acceptance angle, and *i_c_* represents the incidence critical angle (corresponding to a refraction angle of 90°). Ray 3 is totally reflected inside the fiber core and after multiple reflections in the core/cladding boundary emerges at the other side of the fiber.

**Figure 2:**
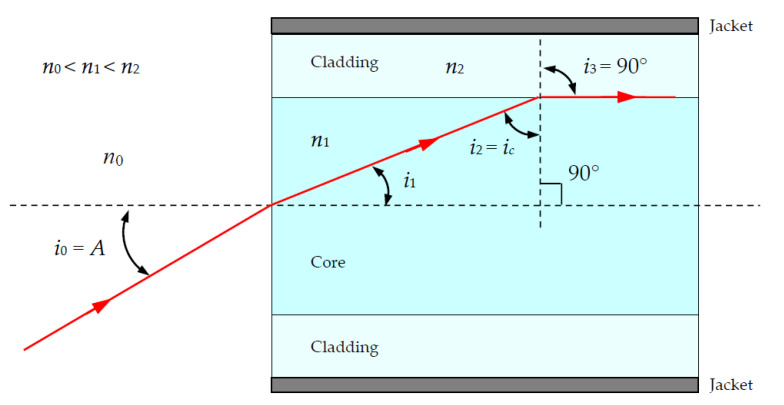
Optical fiber in the case of light rays entering at the limit of acceptance angle *A*.

Figure 2 shows the case of light rays entering at the limit of acceptance angle *A* and critical angle *i_c_* for total internal reflection (TIR) between core and cladding; *n*_0_, *n*_1_, and *n*_2_ are, respectively, the refractive indices of the external medium, the core medium, and the cladding medium, so TIR is only possible if *n*_2_ < *n*_1_.

According to Snell–Descartes’s law [36],

*n*_1_ sin *i*_1_ = *n*_0_ sin *i*_0_ (1)

*n*_1_ sin *i_c_* = *n*_2_ sin 90° = *n*_2_ (2)

and, since *i_c_* = 90° − *i*_1_

sin *i_c_* = cos *i*_1_ (3)

and

*n*_1_ cos *i*_1_ = *n*_2_ (4)

Squaring and adding both Equations (1) and (4), and considering *i*_0_ = *A*, we obtain

*n*_1_^2^ sin^2^
*i*_1_ + *n*_1_^2^ cos^2^
*i*_1_ = *n*_0_^2^ sin^2^
*A* + *n*_2_^2^ (5)

(6) $$\sin A=\sqrt{\frac{n_{1}{}^{2}-n_{2}{}^{2}}{n_{0}{}^{2}}}=\frac{1}{n_{0}}\sqrt{n_{1}{}^{2}-n_{2}{}^{2}}$$

The magnitude *n*_0_ sin *A* is called the numerical aperture (NA). The acceptance light cone is defined by the angle 2*A*. Light rays entering at *i*_0_ > *A* pass to the cladding and will not be transmitted by the optical fiber. Only rays entering at *i*_0_ ≤ *A* will be transmitted. In that case, *i*_2_ ≥ *i_c_*.

Admitting that an optical fiber is used with both ends surrounded by air, the refractive index of air (*n*_0_) at ambient normal conditions (pressure *p*_0_ = 101.325 kPa and temperature T = 20 °C) is about 1.00027 near the visible spectrum (1.000281 for λ = 350 nm, 1.000273 for λ = 550 nm, and 1.000269 for λ = 1000 nm) [37]. Thus, we can consider *n*_0_ ≈ 1, and then NA = sin *A*, and *A* = arcsin $\sqrt{n_{1}{}^{2}-n_{2}{}^{2}}$.

For each optical material, the refractive index (*n*) changes with the wavelength λ (curves of refractive index as a function of wavelength can be consulted in reference [28]), thus changing the limit angle and other key aspects of the behavior of an optical fiber. The refractive index variation is usually well described, particularly in the visible spectrum, by Cauchy’s equation [36]:

*n* = *a* + *b*/λ^2^ + *c*/λ^4^ (7)

where *a*, *b*, *c* are constants, specific for each material.

To compare optical materials, the Abbe number (*V_d_*) [36] is often used:

*V_d_* = (*n*_D_ − 1)/(*n*_F_ − *n*_C_) (8)

where *n*_C_, *n*_D_, and *n*_F_ are the refractive indices measured for three wavelengths covering the visible spectrum and corresponding to the C, D, and F Fraunhofer lines: 656.3 nm (red), 589.3 nm (yellow), and 486.1 nm (blue), respectively.

The higher the Abbe number is, the more constant is the refractive index, a desirable feature particularly in lenses and other sunlight receiving optical devices. The Abbe number for silica glass (~68) or borosilicate BK7 glass (~64) is higher than for commonly used polymers such as PC (~28), PS (~30), or PMMA (~53) [32].

From these theoretical notes, it becomes clear that the global transmission efficiency of a bundle of fibers will depend not only on the individual characteristics of each fiber, but also on the fiber array, and it is supposed to be a function of the incidence angle.

## 3. Experimental

### 3.1. The Optical Fiber Bundle

**Figure 3:**
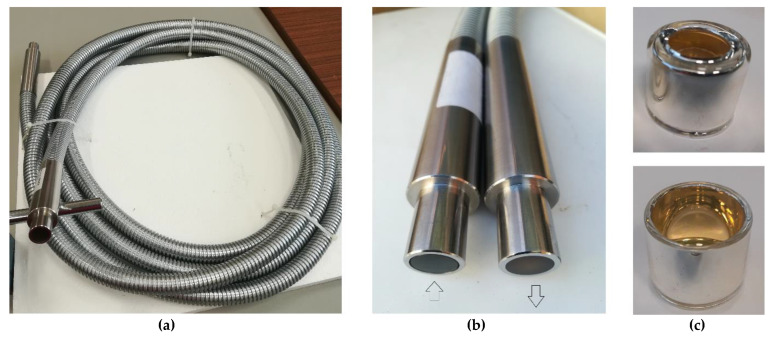
(**a**) Photo of the 7 m optical fiber bundle/cable specially designed to transmit concentrated sunlight (up to 1000 W_th_); (**b**) detail of the terminals (inlet and outlet) of the optical fiber bundle/cable; (**c**) front (above) and back (below) view of the protection cap.

Figure 3 depicts the 7-m-long optical fiber bundle/cable that was tested in this work. The bundle/cable was specially designed and manufactured by Ceram Optec SIA (Riga, Latvia) [38]. The maximum input allowed power is 1 kW_th_ distributed in a circle with 14 mm diameter (the effective optical waveguide diameter), and according to the manufacturer the bundle/cable is composed of 4900 individual fibers with a small diameter to obtain a high-filling-ratio fiber array, i.e., a compact and homogenous fiber array, with minimum dead space between the optical fibers. According to the manufacturer, the optical core of each fiber has a diameter of 179 μm ± 2% and is made of pure fused silica. Each fiber contains two fluorine-doped fused silica claddings, namely cladding 1 (external diameter = 189 μm ± 2%) and cladding 2 (external diameter = 200 μm ± 2%), and a final jacket that gives to each fiber a total diameter of 220 μm ± 2%. The NA value of the fibers is referred to as being 0.22 ± 0.02. Both end faces of the fiber bundle/cable were polished with 0.3 μm diamond paste. In the inlet terminal, the fiber jackets were removed, and fibers were fused to an additional block of pure silica glass (with ~3 mm thickness) to improve the heat resistance at the light entrance.

Due to the way the inlet terminal was designed and protected, the concentrated solar energy was expected to be delivered to the individual fiber cores without significant losses to the cladding matrix forming the cable, so the temperature should not have been raised due to the passage of light (radiation heat losses in the fibers should be negligible). Nevertheless, this cable was designed to withstand a maximum temperature of 400 °C along its total length. To reflect the radiation that could heat up the cable ferrule, made of stainless steel, the entrance of the cable was protected with a cap (see Figure 3c) made of BK7 glass, completely silver plated.

### 3.2. Solar Testing Facility

**Figure 4:**
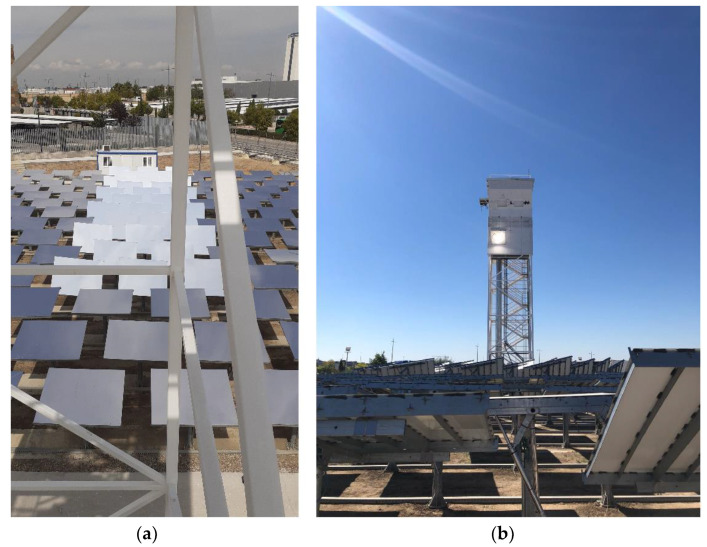
Images of the Very High Concentration Solar Tower (VHCST) facility: (**a**) Selection of heliostats used in the experimentation; (**b**) solar tower, in which the testing zone is pointed out by the concentrated solar light spot.

The optical fiber bundle/cable was installed at the Very High Concentration Solar Tower (VHCST) facility, at IMDEA Energy (Móstoles, Madrid, Spain). The VHCST has a customized heliostat field composed of 169 single-facet tilt–roll heliostats of 3 m^2^ (1.6 m × 1.9 m) with focal lengths of 20 and 30 m. Further details about this testing facility can be found in works by Romero et al. [39,40] and Martínez–Hernández et al. [41]. Due to the low acceptance angle of the optical fiber cable, only some selected heliostats of the VHCST facility were used in this analysis (see Figure 4 and Section 3.4).

### 3.3. Experimental Layout, Sensors, and Other Apparatuses Used in the Tests

During the solar irradiation tests conducted at the VHCST, the following equipment was used:

1. a pyrheliometer (SHP1-A, Kipp and Zonen, Delft, The Netherlands) mounted on a sun tracker, to monitor the direct normal irradiance (DNI) reaching the heliostats;
2. two Gardon-type radiometers (Vatell Circular-Foil Heat Flux Transducer TG1000-1, Vatell Corporation, Christiansburg, VA, USA) to measure the input and output radiation flux of the optical fiber bundle/cable;
3. a water chiller (model Huber Unichiller 003-MPC, Huber Kältemaschinenbau AG, Offenburg Germany) to lower the temperature of the Gardon-type radiometers;
4. a white screen made of plates of alumina to protect the testing room at the VHCST from the effects of the concentrated solar radiation;
5. a CCD camera and software (GT1930L, Allied Vision, Stadtroda, Germany) to monitor the distribution of radiation flux over the white screen;
6. several K-type thermocouples (TC Ltd., Uxbridge, UK) to monitor the temperature at different locations over the white screen, as well as the temperature close to the entrance of the optical fiber bundle/cable;
7. an optical converging system to re-concentrate the radiation at the outlet of the cable. The converging system (constructed at Instituto Superior Técnico, Lisbon, Portugal) is composed of two identical plano-convex lenses made of silica glass, as described in the work of Rosa et al. [42];
8. a LabVIEW system for data acquisition and monitoring of temperature and radiation flux along time.

**Figure 5:**
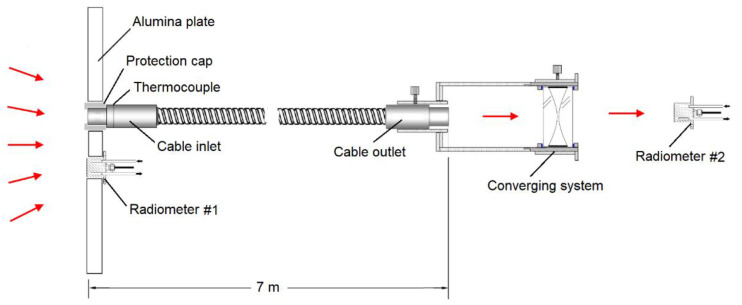
Diagram explaining the experimental layout used in this work.

Figure 5 sketches the experimental layout used in this work. Radiometer #1 was placed near the cable inlet, and radiometer #2 was placed after the converging system. The ratio between the values provided by these radiometers gave us directly the efficiency of the optical cable. 

**Figure 6:**
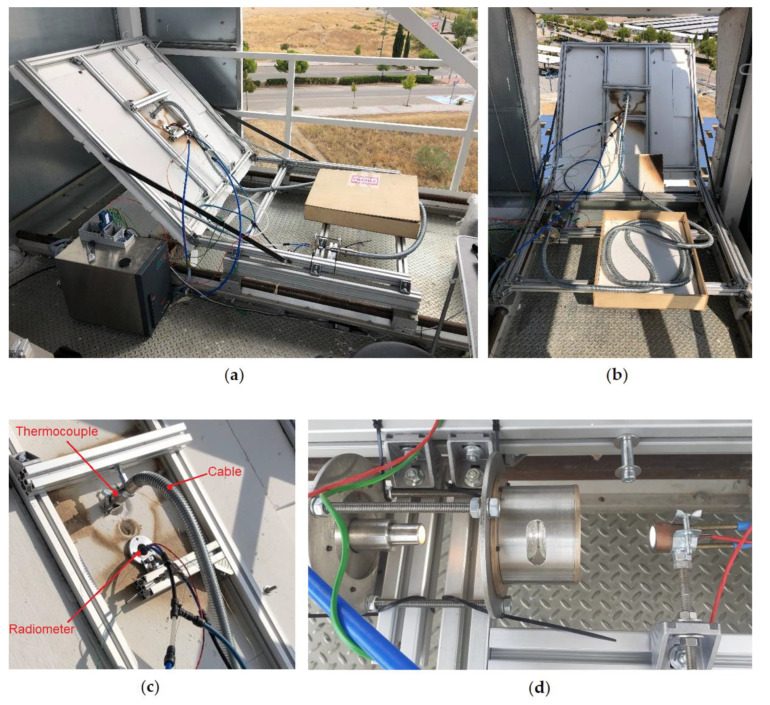
Images of the testing room at the solar tower: (**a**) General layout used for testing the optical fiber bundle/cable; (**b**) cable ready to be irradiated; (**c**) positions of the radiometer #1 and thermocouple at the entrance of the cable; (**d**) converging system and radiometer #2 in front of the outlet of the cable, during an irradiation test.

Figure 6 shows images of the testing room at the solar tower. Details of the positions of the Gardon-type radiometers used to measure the radiation flux at the entrance (radiometer #1) and at the outlet of the cable (radiometer #2) are shown in Figure 6c,d.

The radiometer #2 used to measure the radiation flux at the outlet of the cable was positioned symmetrically relatively to the center of the converging system, thus allowing us during an irradiation test to obtain a well-focused light beam, as is shown in Figure 6d. 

**Figure 7:**
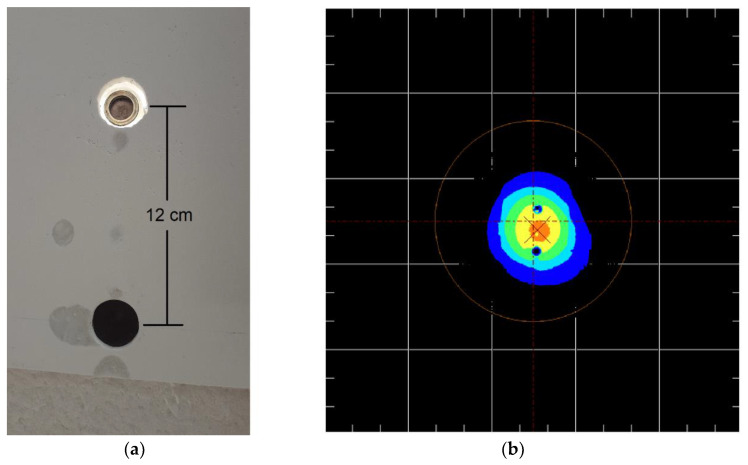
(**a**) Entrance of the cable (above) and radiometer #1 (below) at the irradiated surface of the alumina plate; (**b**) radiation flux map obtained with the imaging software using the CCD camera; the black spot above is the entrance of the cable, and the black spot below is the radiometer #1.

Due to physical constraints, radiometer #1, used to monitor the radiation flux at the inlet of the cable, was positioned at the irradiated alumina surface at a 12 cm distance from the entrance of the cable (see Figure 6c and Figure 7a). To obtain the same irradiance at the radiometer and bundle entrance, the heliostats were directed towards the middle point between both locations. During the irradiation tests, irradiance on the alumina plate was continuously monitored using an indirect method with the CCD camera. This technique also allowed for improved heliostats pointing and the performing of corrections to detect deviations of the heliostats. Figure 7b is an image provided by the camera software during a test showing the distribution of radiation flux over the irradiated area of the alumina screen. It shows that the radiation flux was approximately equal for the positions indicated by the two black spots.

### 3.4. Testing Procedure

**Figure 8:**
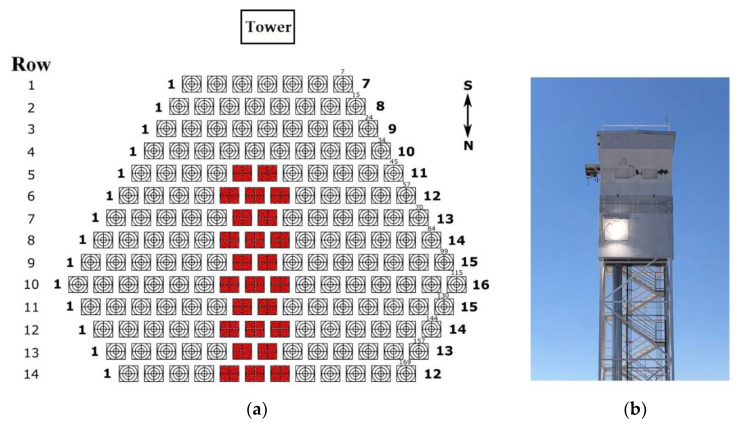
(**a**) Heliostat field layout of the VHCST facility. The heliostats used in the tests are marked in red; (**b**) illuminated alumina screen where the entrance of the optical fiber cable was positioned together with a Gardon-type radiometer (radiometer #1).

Figure 8 shows the heliostats used in the tests and the illuminated alumina screen seen from the heliostat field. Only two or three heliostats were employed per row, with those more centered towards the alumina screen (the target). First, the two heliostats at row 5 were used, because they provided an incident radiation beam more perpendicular to the entrance of the cable. These two heliostats at row 5 were then removed and replaced by the three heliostats at row 6, used to focus the sunlight onto the aiming points located into the target area. As is further explained later, the radiation beam provided by the three heliostats at row 6 is less perpendicular (8.9°) than the beam provided by the heliostats at row 5 (4.5°). In this way, this procedure was repeated till row 14, which provides a much more inclined beam of radiation (25.7°).

**Figure 9:**
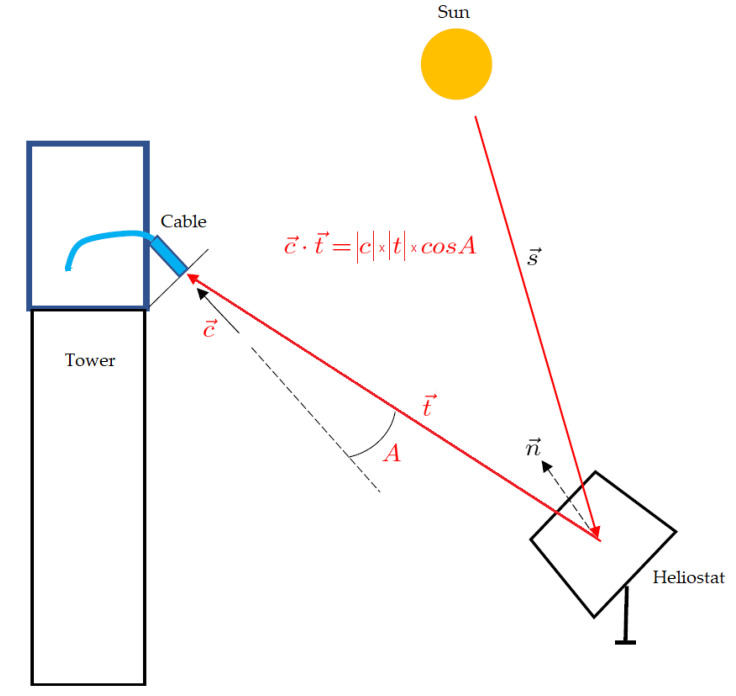
Model for calculating the angle of incidence (*A*) for the radiation coming from a single heliostat.

The angle of incidence for the radiation coming from a single heliostat was calculated assuming that the radiation comes from the heliostat center and points to the target point in the tower, where the cable entrance was installed, as shown in Figure 9. 

As the cartesian coordinates of each heliostat center and of the target point are well known, we can work out the light vector *t* going from the heliostat to the target point (see Figure 9). The cable entrance was installed precisely at the target point, along a carefully measured direction, defined by a vector *c*. The dot product of these two vectors, *c·t*, gives the incidence angle *A* of the incident light entering the cable. According to their position in the heliostat field, groups of 2 or 3 heliostats were selected from the same row in order to have approximately the same aperture angle *A*. The final values, reported in Table 2, were obtained, averaging the incidence angles for the various heliostats involved in each measurement.

## 4. Results and Discussion

Due to the very low number of heliostats used for each group/row (only two or three heliostats, as shown in Figure 8) and the light focusing conditions attained during each irradiation test (Figure 7b), the values of radiation flux at the entrance of the cable could not be very high (maximum attained value was 144.8 kW/m^2^). Table 3 summarizes the data obtained from the irradiation tests conducted with the different groups of heliostats used in the calculations of the transmission efficiency of the cable as a function of the incidence angle. For each group/row of heliostats (from row 5 to row 14), and assuming the same irradiance at radiometer #1 and the cable entrance, Table 3 shows the values of radiation flux measured by the radiometers #1 (flux in) and #2 (flux out).

The values of direct normal irradiation (DNI), in W/m^2^, also shown in Table 3, were measured by a pyrheliometer (model SHP1-A from Kipp and Zonen) mounted on a sun tracker (placed near the center of the heliostat field). Due to weather conditions, DNI values may have changed during the irradiation tests, but that did not affect the attainment of the objective of this work: the determination of the efficiency of the cable transmission (calculated as the ratio obtained dividing the flux measured at the exit by the flux measured at the entrance of the cable).

The protection system or thermal shielding (dense alumina plate plus highly reflective protection cap) put in place to avoid the concentrated radiation heating up the non-optical parts of the cable (specially the metallic ferrule at the entrance) proved to be adequate. During the whole irradiation testing program, the maximum temperature reached at the inlet ferrule of the cable (measured by the thermocouple shown in Figure 6c) was 60 °C.

**Figure 10:**
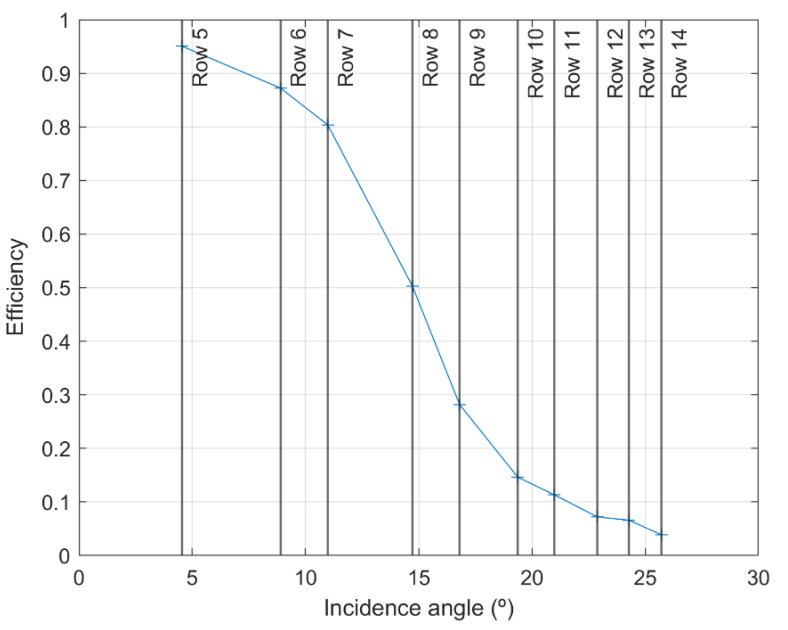
Efficiency of the optical fiber cable as a function of the incidence angle.

The transmission efficiency of the optical fiber cable as a function of the incidence angle is plotted in Figure 10. The transmission efficiency (or average transmissivity) of the fiber cable varied from a very large value (circa 95%) for an incidence angle of circa 4.5°, to a very low value (<5%) for an incidence angle of circa 25.7°.

If the value of 4.5° is assumed as an average value for the acceptance angle of the optical cable, then considering NA = sin *A*, NA = 0.08 is obtained. However, if it is assumed that *A* = 14.7° (corresponding to 50% efficiency in Figure 10) this results in NA = 0.25, a value close to the NA value of the individual fibers, indicated by the company that manufactured the fibers and the cable, which was NA = 0.22 ± 0.02. Theoretically, a NA = 0.22 corresponds to an acceptance angle of 12.7°, but due to the ±0.02 uncertainty, the angle will be between 11.5° and 13.9°.

When the angles of incidence are low (<~15°), light losses in this type of cable inlet are very small. Consequently, the system used to protect the entrance of the cable was revealed to be adequate, not significantly affecting the amount of radiation power entering the cable.

On the other hand, it is not expected to reach high temperatures inside the cable, and therefore influence of temperature on the refraction index of silica glass is not foreseen as a significant issue. The air refractive index in turn changes very little with temperature, decreasing from n = 1.0002736 at 15 °C to *n* = 1.0001959 at 100 °C (for λ = 1052 nm, 50% humidity and 450 ppm CO_2_) [37].

## 5. Conclusions

A 7-m-long optical fiber bundle/cable with an effective aperture circular area of 14 mm diameter, specially designed and manufactured by a leading company to transmit up to 1000 W_th_ of unfiltered concentrated sunlight, was tested at the receiver position of a very high-concentration solar tower.

The testing methodology based on using different selected heliostat groups of the VHCST facility proved to be suitable to determine the efficiency of the optical fiber cable in the function of the incidence angle. The average transmissivity of the cable was higher than 50% if the incidence angle of the solar radiation was lower than 14.7° and reached 95% when the incidence angle was lower than 4.5°, proving that heat was efficiently transmitted through the optical cable bundle when radiation was properly injected into the cable. The cable withstood these tests without revealing any type of damage. This work validates future research towards the use of advanced fused-silica-core optical fibers in solar-to-heat conversion technologies.

Finally, it should be outlined that the herein described test set-up, and its corresponding methodology, to determine the transmission efficiency of the fiber cable was used simply because with other type of solar concentrated facilities (such as parabolic concentrators, point-focusing Fresnel lenses, etc.), this kind of measurements seemed not possible. Once we could only use two or three heliostats for setting each average incidence angle, the values of radiation flux at the entrance of the cable were very far from the expected capacity of the cable. In our experiments, the maximum attained value of flux at the entrance of the cable was only 14.48 W/cm^2^ (i.e., 144.8 kW/m^2^), but the full capacity of the cable was circa 650 W/cm^2^ (if we assume 1000 W distributed in a circle with 14 mm diameter, which is the effective optical waveguide diameter of the cable). Therefore, further studies for the continuation of this research line should be dedicated to the assessment of the maximum power transmission capacity of the cable, once we can develop a new test set-up allowing a much higher concentration of unfiltered sunlight, adequately provided with low incidence angle.

## Figures and Tables

**Table 1:** Thermal and optical properties of materials used to fabricate optical fibers. (Values indicated in the table were extracted from two databases: [31,32]).

Material	Glass Temperature [31] (°C)	Maximum Service Temperature [31] (°C)	Coefficient of Thermal Expansion [31] (10^−6^/°C)	RefractiveIndex * [32]	Abbe Number [32]
Silica glass	957–1560	897–1400	0.55–0.75	1.45	67.8
Polymethyl methacrylate (PMMA)	85–165	42–57	72–162	1.48	53.2
Polystyrene (PS)	74–110	77–103	90–153	1.57	29.5
Polycarbonate (PC)	142–205	101–144	120–137	1.57	27.9

* measured at λ = 1052 nm wavelength.

**Table 2:** Average values of angle of incidence for the 2 or 3 heliostats used in each row.

Heliostat Row	5	6	7	8	9	10	11	12	13	14
**Angle (°)**	4.5	8.9	11	14.7	16.8	19.4	21	22.9	24.3	25.7

**Table 3:** Summary of data obtained from the irradiation tests, used to determine the transmission efficiency of the optical cable.

Heliostat Row	Angle (°)	DNI (W/m^2^)	Flux In (kW/m^2^)	Flux Out (kW/m^2^)	Efficiency (%)
5	4.5	708	28.2	26.9	95.1
6	8.9	747	75.2	65.6	87.2
7	11	718	73.7	59.3	80.4
8	14.7	725	144.8	72.8	50.3
9	16.8	711	31.8	9	28.2
10	19.4	674	52.9	7.7	14.6
11	21	552	36.7	4.2	11.3
12	22.9	456	32.4	2.3	7.2
13	24.3	649	37	2.4	6.6
14	25.7	696	43.8	1.7	3.9

## Data Availability

Data sharing is not applicable to this article.
